# The role of L1-L2 dissimilarity in L2 segment learning – Implications from the acquisition of English post-alveolar fricatives by Mandarin and Mandarin/Wu speakers

**DOI:** 10.3389/fpsyg.2022.1017724

**Published:** 2022-12-13

**Authors:** Wenjun Chen, Jeroen van de Weijer

**Affiliations:** ^1^School of Foreign Languages, Ningbo University of Technology, Ningbo, Zhejiang, China; ^2^College of International Studies, Shenzhen University, Shenzhen, China

**Keywords:** post-alveolar fricatives, Mandarin Chinese, Wu, L1-L2 dissimilarity, learning results

## Abstract

This study examines how the concept of L1-L2 dissimilarity should be addressed from a two-way perspective in L2 segment learning, and how it relates to the learning outcomes. We achieved this by investigating the productions of the post-alveolar fricatives /ʃ, ʒ/ by Mandarin and Mandarin/Wu speakers, which were subsequently assessed by native English listeners. In the first experiment, we analyzed the spectral moments of /ʃ, ʒ/ produced by Mandarin monolingual and Mandarin/Wu bilingual speakers to find out how the two groups of speakers pronounced the target segments. In the second experiment, native English listeners were tasked with rating the accentedness of the Mandarin- and Mandarin/Wu-accented /ʃ, ʒ/. Results showed native English listeners scored Mandarin/Wu-accented /ʃ/ as having no accent and Mandarin-accented /ʒ/ as having a heavy accent, indicating that English natives perceived the ‘native vs. nonnative’ segment dissimilarity differently from Chinese learners of English, and that the L1-L2 dissimilarity perceived from both sides may work together in defining the L2 segment learning outcomes.

## Introduction

### Learning of L2 sounds

Learning a second language (L2) past a certain age can be a challenging task that takes a lot of time and effort. Most L2 learners will have to learn to process sounds that are absent or not contrastive in their native language. In fact, infants are able to discriminate almost any speech sound distinction at an early stage (Werker, 2018; Reh et al., 2021), but as they age neurologically, their sensitivity to non-native sounds declines, possibly resulting in generating the L2 (second language) sounds with accents associated with their L1 (Flege et al., 1999; Edwards and Zampini, 2008; Saito et al., 2020). This has led to the development of theories on non-native speech perception and production.

Most assumptions about non-native speech perception and production view L1 (the first language) phonetic inventory as a ‘filter’ on L2 (the second language) phonetic learning (Georgiou, 2018; Kilpatrick et al., 2019). The essence of this idea has been reflected in non-native speech learning theories/models, including the ‘Native Language Magnet model’ (NLM; Kuhl, 1991, 1992; Kuhl and Iverson, 1995), the ‘Perceptual Assimilation Model’ (PAM; Best, 1994; Best and Tyler, 2007; Best et al., 2019; Li et al., 2021), and the ‘Speech Learning Model’ (SLM; Flege, 1988, 1992, 1995; Flege et al., 2003; Flege and Bohn, 2021), etc. The central idea of these theories is that the perceptual and production space of L2 segments can be altered by the L1 phonetic categories of the learners, so the learning outcomes are not always desirable (Chang, 2019). Among others, two theoretical frameworks, namely the Speech Learning Model (SLM) and the Perceptual Assimilation Model-L2 (PAM-L2), relate non-native segment learning to learners’ L1 phonetic inventory by incorporating multiple factors like L2 experience, L2 production and perception, L2 phonetic categories, etc., that are critical to the learning outcome.

The SLM theory seeks to account for variations in the extent to which L2 learners are able to form a new L2 phonetic category (i.e., vowels or consonants) by considering the perceived distance between a target L2 phone and its L1 equivalence. In its early version (Flege, 1995), SLM proposed that humans’ ability to form new phonetic categories remains intact and accessible across the life span. A new phonetic category will be formed for an L2 sound when learners discover (or discern) phonetic dissimilarities between the L2 sound and the L1 sound(s) that is (are) closest in phonetic space, and that learners would be more successful in learning ‘new’ rather than ‘similar’ L2 segments. The reason is that the L2 sounds that are similar to L1 counterparts are mapped onto (or: assimilated to) these L1 sounds, so small deviations from the L2 to the L1 sound are not easily perceived by the language learners. In contrast, sounds that are dissimilar from any L1 sound are not assimilated and thus the L2 category can be more successfully formed (Flege, 1995). Over the years, SLM has continued to be developed (Flege, 1988, 1992, 1995; Flege et al., 2003; Flege and Bohn, 2021). The latest version of SLM-r (2021) revisited and modified some of the previous claims, like redefining the L2 experience, time of exposure to L2, and incorporating inter-subject variability, raising the ‘perceived distance’ measurement question, and thus provided a framework for research that may eventually permit a better understanding of how speech is learned across the life span. Despite these adjustments, it remains based on the idea that the greater the perceived dissimilarity of an L2 segment from the closest L1, the more likely it is that a new category will be formed for the L2 sound.

The original PAM theory, on the other hand, was primarily concerned with the development of L2 categories by naïve L2 learners. It claims that when listening to an unfamiliar, nonnative phone, naïve listeners would perceive it as a good or poor exemplar of a native phonological segment (Categorized), or as unlike any single native phoneme (uncategorized), or rarely, as a non-linguistic nonspeech sound (Non-Assimilated; Best, 1994). PAM later extended its scope from ‘naïve listeners’ to ‘L2 speech learners’, contrasting its own framework and postulates with those of SLM. The modified PAM (as PAM-L2; Best and Tyler, 2007) seeks to analyze the perceived L1-L2 dissimilarity at both the phonetic and phonological levels (rather than just the phonetic level, as handled by SLM) and predicts success at L2 perceptual acquisition across a variety of scenarios: (1) when only one L2 phonological category is perceptually assimilated to a given L1 phonological category, (2) when both L2 phonological categories are perceptually assimilated to the same L1, with equally or unequally good instances of that category, (3) when there is no L1-L2 phonological assimilation. Although the two theories differ in many respects, there are some overlaps in their underlying logic. For example, when both L2 categories are perceived as equivalent to the same L1 category, but one is perceived as being more different than the other, PAM-L2 predicts that a new L2 category will be formed for the deviant L2 phone (Best and Tyler, 2007), which endorses the rationale behind the SLM theory in that the formation of a new phonetic category for an L2 sound depends on the sound’s degree of perceived dissimilarity from the closest L2 sound.

SLM has been successful in characterizing the learning of L2 sounds in various studies (Flege et al., 1996; McAllister et al., 2002; Georgiou, 2021; Strandberg et al., 2021; Yang et al., 2022); nevertheless, exceptions are still occasionally noticed. Some studies demonstrate that degrees of L1-L2 dissimilarity do not always indicate how well the L2 segments are learned (Wester et al., 2007; Iverson et al., 2008; Schmidt, 2018; Yang, 2019), and that assessing the perceived L1-L2 dissimilarity can be challenging and thus produce contradictory results (Cebrian, 2006; Iverson and Evans, 2007). In fact, a standard measurement for the perception of L1-L2 dissimilarity has yet to be validated (Flege and Bohn, 2021). However, a more important concern about this ‘L1-L2 dissimilarity’ argument is how it is involved in defining L2 learning success. SLM relates L2 learners’ perceived L1-L2 distance to learning difficulties, which in turn predicts the ultimate learning result (Flege, 1995; Flege and Bohn, 2021). But ‘learning difficulty’ and ‘learning result’ can be two interrelated but relatively independent factors (Scheffler, 2011). ‘Learning difficulty’ can be reasonably described from the perspective of L2 learners, whereas ‘learning result’ is more of an objective matter that should be evaluated based on native listeners’ (fluent speakers’) judgments (Silva and Roehr-Brackin, 2016; Birdsong, 2021). Similar to L2 learners’ perceived L1-L2 dissimilarity, native listeners’ perceived distance between the ‘non-native substitute (produced by L2 learners)’ and ‘native segment’ may also play a significant role in determining the learning result. As a matter of fact, the idea of ‘similarity or dissimilarity’ can often be influenced by perspectives: L2 learners and native listeners may perceive it in different ways (i.e., L1-L2 dissimilarity for L2 learners, or analogously, ‘non-native substitute—native segment dissimilarity’ for native listeners), depending on how their L1 phonetic inventory and phonological systems differ (Best and Tyler, 2007; Hyman, 2018; Moran, 2019). For example, SLM predicts that a small L1-L2 dissimilarity perceived by L2 learners might result in failure of L2 category formation: Specifically, the L2 learner may possibly produce the target segment with strong characteristics similar to the closest L1 counterpart. However, native listeners of this language may find this non-native substitute fairly acceptable and thus rate it as accentless, because it is close to the target sound from their perspectives as well. On the other hand, if the native speakers are rather sensitive to the non-native substitute and take it as quite distinct from any segment in the L2 phonetic system, they would rate this production as having a severe accent.

In light of the foregoing, the current study aims to investigate how learners with different L1 backgrounds can anticipate their learning outcomes for the L2 segments. We gather data on how native speakers evaluate the learning outcomes of L2 segments, as well as the factors that may influence that evaluation. To achieve this, we investigate the production of the English post-alveolar fricatives /ʃ, ʒ/ by Mandarin and Mandarin/Wu speakers. The outputs of each group are then evaluated by native English listeners to identify any differences. Because Mandarin and Wu both have their own post-alveolar fricatives that are similar to, but not identical to English /ʃ, ʒ/, one may thus predict that learning results for the target sounds differ between Mandarin and Mandarin/Wu speakers, due to the alleged difference in L1-L2 dissimilarity. Thus, Mandarin or Mandarin/Wu speakers’ distinct perspectives on the ‘L1-L2 post-alveolar fricative dissimilarity’ may provide us with an opportunity to examine how different L2 outputs can be achieved in line with it.

### Chinese post-alveolar fricatives

Chinese is a group of related but in many cases mutually unintelligible language varieties. The largest dialect group is Mandarin, and the so-called Standard Chinese is a standardized form of the Beijing variety of Mandarin. Another large dialect group of Chinese is Wu, whose phonological system differs drastically from that of Mandarin. Within Wu, there are several varieties, which are not (entirely) mutually intelligible (Wang, 2014). The younger generations of Wu speakers (under 35 years old) are mostly Mandarin/Wu bilinguals (Zhong and Chen, 2012), they have been exposed to Wu since birth and regularly to Mandarin Chinese from 3 to 4 years in nursery school. These Chinese speakers started learning English in middle school at an average of 13 years old.

English, Mandarin and Wu differ in their inventories of post-alveolar fricatives (Table 1). /ʃ/ and /ʒ/ are vocally contrasted in English; similarly, Wu has this contrast between/ɕ/ and /ʑ/ (/ʑ/occurs in many subdialect regions in the northern part of Zhejiang province, like Shaoxing, Ningbo, Taizhou, etc.). Mandarin, on the other hand, has a voiceless palatal /ɕ/ and two retroflexes /ʂ, ɻ/. All these segments are fricatives generated in the post-alveolar region, but they differ in degrees of palatalization. For example, /ɕ/ (Mandarin or Wu) and /ʑ/ (Wu) are fully palatalized with the tongue blade rising towards the hard palate and the middle of the tongue curving and pointing upward (Pan et al., 1991; Jacques, 2006). English /ʃ, ʒ/ are partially palatalized with the tongue blade behind the alveolar ridge and the front of the tongue bunched up to the palate (Gussenhoven and Aarts, 1999; Zharkova, 2019). By contrast, the retroflex /ʂ, ɻ/ are produced by raising the apical or laminal part of the tongue toward the hard palate, which generates hardly any palatalization (Maddieson and Ladefoged, 1996).

**Table 1:** Comparison of fricatives produced in the post-alveolar region in English, Mandarin and Wu.

	Post-alveolar	Retroflex	Alveolo-palatal
Mandarin		/ʂ/, /ɻ/	/ɕ/
Wu			/ɕ/, /ʑ/
English	/ʃ/, /ʒ/		

Phonologically, English /ʃ/ and /ʒ/ are [−anterior] [+distributed] in terms of place feature, the only difference between the two is the laryngeal feature [±voiced] (Kenstowicz, 1994). The fricatives /ɕ/ and /ʑ/ in Wu have the same contrastive features ([±voiced, −anterior, +distributed]) as English, whereas the Mandarin pair /ʂ, ɻ/ has the features of [±voiced], [−anterior], and [−distributed] (Duanmu, 2007; Table 2).

**Table 2:** Phonological features of the post-alveolar fricatives in English, Mandarin and Wu.

	Anterior	Distributed	Voiced	Approximant
English/ʃ/	−	+	−	−
English /ʒ/	−	+	+	−
Mandarin/ʂ/	−	−	−	−
Mandarin/ɻ/	−	−	+	+
Mandarin/Wu /ɕ/	−	+	−	−
Wu /ʑ/	−	+	+	−

The phonological feature ‘anterior’ can be used to distinguish between segments made with the tongue tip at the front of the mouth ([+anterior]) and those that are not ([−anterior]); on the other hand, the feature ‘distributed’ is employed to differentiate between segments that employ only the tip of the tongue for articulation ([−distributed]) and those where the tongue is bunched up in a wide posture ([+distributed]). English, Mandarin, and Wu all contrast their coronal sounds by these two place features (in addition to the feature [±voiced]).

In terms of distribution, English /ʃ/ comes before either front or back vowels, such as /i, u, a/, while English /ʒ/ usually precedes /u, ǝ/ and does not occur word-initially very often. Mandarin /ɕ/can only be followed by the high front vowels /i, y/, whereas /ʂ/does not occur before these two vowels (Duanmu, 2007).

Examples of English words containing /ʃ, ʒ/ and Chinese words containing /ɕ, ʂ, ɻ/ are given in (1, 2):

(1)she /ʃi:/    vision /‘vɪʒən/

  sharp /ʃɑ:p/    usual /‘ju:ʒuəl/

  shoes /ʃu:z/     genre /‘ʒɑ:nrə/

(2)希 /ɕi/ ‘hope’    师 /ʂɨ/ ‘master’   日 /ɻɨ / ‘sunset’

  虚 /ɕy/ ‘empty    书 /ʂu/ ‘book’    如 /ɻu/ ‘if’

  沙 /ʂa/ ‘sand’ 饶 /ɻau/ ‘forgive’

The voiceless alveolo-palatal /ɕ/ in Wu has the same distribution as in Mandarin, while its voiced counterpart /ʑ/ is devoiced in word-initial position, and only voiced intervocalically (as in the example below). Initial devoicing in the Wu dialect is an ongoing change well documented by many researchers (Zhao, 1956; Pan et al., 1991; Shen, 2020). In an investigation of voiced segments produced by the younger generation of Mandarin/Wu dialect speakers (under 35), Zhong and Chen (2012) found that many voiced consonants, including /ʑ/, were devoiced even in the intervocalic position. This phenomenon possibly indicates a transfer from Mandarin to Wu, as there are no voiced fricatives in Mandarin.

(3) 树 /ʑy/ ‘tree’ (devoiced [ʑ])

桃树  /tau ʑy/ ‘peach tree’ (voiced [ʑ])

Another point that deserves attention is the phonological status of Mandarin /ɻ/. It is produced by narrowing the vocal tract with the subapical part of the tongue, but usually not enough to create the turbulent airstream characteristic of fricatives. However, the amount of turbulent airstream produced in this way varies among Mandarin speakers (Lee and Xue, 2011), so this Mandarin retroflex is represented by the IPA symbols /ɻ/~/ʐ/. The status of the sound has been a topic of debate for many years. Some scholars (Li, 1983; Zongji and Maocan, 1989; Lin, 2001) asserted it to be a fricative for its obvious narrow constriction point (demonstrated by electropalatography) and the trace of noise found in the spectrogram; Others (Zhu, 2007; Fu and Monahan, 2021) argued that /ɻ/ should be considered an approximant, on the basis of its shorter duration and weaker amplitude than that of other fricatives such as /s, ʂ/. Zhu claimed that frication noise could be observed in the spectrogram of this sound, but the amount of noise was less than that of the sibilants /s/, /ʂ/, and /ɕ/. Nevertheless, in the current study, we measured Mandarin /ɻ/, phonetically, as a fricative, due to its acoustic character, while phonologically we viewed it as an approximant.

In the following sections, we investigate the learning of English post-alveolar fricatives /ʃ, ʒ/ by Mandarin and Mandarin/Wu speakers. First, we measure Mandarin and Mandarin/Wu speakers’ production of the target sounds using acoustic analysis. Then native English listeners’ judgments on these productions are obtained to examine how well they are generated. In order to achieve this, a production experiment and a rating test are conducted to gather information regarding L2 fricative production and the native listeners’ evaluation. Here, we anticipate that the two Chinese groups would produce the target English segments /ʃ, ʒ/ showing characteristics of their respective L1s (Best and Tyler, 2007; Flege and Bohn, 2021). Specifically, Mandarin has the voiceless post-alveolar /ʂ/ or /ɕ/, and the voiced post-alveolar /ɻ/, so Mandarin monolinguals might produce English voiceless /ʃ/ exhibiting phonetic characteristics of /ʂ/ or /ɕ/, and the English /ʒ/ with features of Mandarin /ɻ/. On the other hand, since the L1 system of Mandarin/Wu bilinguals involves two sets of post-alveolar fricatives, i.e., one from Mandarin (voiceless /ʂ/ or /ɕ/ vs. voiced /ɻ/) and one from Wu (voiceless /ɕ/ vs. voiced /ʑ/), their expected English productions are more difficult to predict. However, as voiceless /ɕ/ appears in both Mandarin and Wu, this sound may be more likely to surface than the Mandarin-specific voiceless /ʂ/ in these bilinguals. Therefore, as a tentative prediction, we expected that Mandarin/Wu speakers would produce English /ʃ/ with traits of the voiceless /ɕ/. By contrast, the bilinguals would produce the target /ʒ/ exhibiting features of either Mandarin /ɻ/ or Wu /ʑ/, i.e., the L1 counterparts of the voiced post-alveolar /ʒ/ in English. Finally, we speculated that some production types might be more accented to native English listeners than others, so the evaluation of accentedness in various L2 outputs might differ.

## Production experiment

The aim of the current experiment is to investigate the English post-alveolar fricatives /ʃ, ʒ/ produced by Mandarin- and Mandarin/Wu speakers. An acoustic analysis of the fricatives is performed to achieve this goal.

### Methods

#### Participants

Participants were 30 Chinese speakers, who were split into two groups based on the languages they were exposed to from an early age: one group consisted of 15 Beijing Mandarin speakers (8 males, 7 females) who were all born and raised in Beijing and whose relatives were also Beijing locals, thus these speakers did not speak any dialects other than Mandarin. The second group contained 15 Mandarin/Wu speakers (8 males, 7 females) who were born and raised in the Shaoxing district of Zhejiang province, where the phoneme /ʑ/ appeared in the local dialect (Min et al., 1986). Mandarin/Wu participants had been exposed to the Wu dialect since birth and also to Mandarin Chinese from 3 to 4 years old (in nursery school). They claimed to regularly communicate with their relatives (also Shaoxing locals) in Wu and spoke Mandarin with friends or on formal occasions. All Chinese participants were college students, ranging in age from 19 to 23, non-English majors. Their National Mandarin-speaking qualification test results were all the same (Level II, Grade A), but their CET-4 scores (a National English proficiency test) revealed varying levels of English proficiency (Mandarin participants: mean score = 509, SD = 51; Mandarin/Wu participants: mean score = 518, SD = 47). However, there was no statistically significant difference between the two groups in terms of English level. A control group of 15 native English speakers (8 males, 7 females) also participated in the experiment. These participants were recruited from different universities in Ningbo City (from foreign exchange students enrolled at these universities). Prior to conducting the experiment, the Ethical Committee of the Ningbo University of Technology gave permission for the study, and participants were properly informed of the content and potential risks of the experiment. No participant reported any hearing, visual or speech impairment.

#### Stimuli

The stimuli consisted of 18 words with initial sibilant-vowel (−consonant) sequences. We chose words in English, Mandarin and Wu where the target fricatives were followed by comparable vowels. Specifically, we included the high front vowel /i-ɨ/, the low back vowel /a/, and the high rounded vowel /u-y/ in the reading list. Due to Chinese phonotactic restrictions (Duanmu, 2007), /i/ cannot follow Mandarin /ʂ, ɻ/. We therefore used /ɨ/ instead, as it shares the same articulation place as /i/. For the same reason, the vowel /y/ was used in place of /u/ after the consonant /ɕ/. A total of 18 stimuli were selected (Table 3).

**Table 3:** List of stimuli.

Language	Words					
English	she	/ʃi:/	sharp	/ʃɑ:p/	shoes	/ʃu:z/
	vision	/ˈvɪʒən/	genre	/ˈʒɑnrə/	usual	/ˈjuʒuəl/
Mandarin	师	/ʂɨ/	沙	/ʂa/	书	/ʂu/
	日	/ɻɨ/	扰	/ɻau/	如	/ɻu/
	希	/ɕi/			虚	/ɕy/
Wu	希	/ɕi/			虚	/ɕy/
	(我)己	/ʑi/			(大)树	/ʑy/

The stimuli were embedded in carrier sentences in English, Mandarin and Wu, so that reading lists in all three languages were created.

1. The English carrier sentence ‘Say__ again’ contained consonant-vowel (−consonant; CV or CVC) syllables. The target fricative /ʃ/ appeared in the word-initial position, followed by each of the three vowels /i, u, a/, and the target /ʒ/ was followed by /ə, u, a/. Each stimulus occurred three times, yielding a total of 2 (fricatives) × 3 (vowels) × 3 (times) = 18 tokens. All English, Mandarin, and Mandarin/Wu speakers were invited to produce sentences carrying the English tokens.
2. The Mandarin fricatives /ɕ, ʂ, ɻ/ were embedded in CV syllables in the Mandarin carrier sentence “我说___这个字” (/wo ȿuo/___ /tȿə kə tsɨ/), meaning “I say the word ____”. The fricatives /ʂ, ɻ/ were followed by /ɨ, u, a/, and /ɕ/ by /i, y/. With three repetitions, the Mandarin reading list yielded [2 (fricatives) × 3 (vowels) +1 (fricative) × 2 (vowels)] × 3 (times) = 24 tokens. The carrier sentences embedded with Mandarin tokens were to be spoken by Mandarin and Mandarin/Wu speakers.
3. The fricatives /ɕ, ʑ/ were embedded in the same carrier sentence “我说____这个字” (/ŋo wo/____ /gə ɦəʔz/) articulated in Shaoxing dialect. These fricatives were followed by /i, y/, and occurred in non-word-initial position. In sum, we arrived at 2 (fricatives) × 2 (vowels) × 3 (times) = 12 tokens. These tokens were to be spoken only by Mandarin/Wu speakers.

In total, 45 individuals contributed 1710 tokens, including 810 in English, 720 in Mandarin, and 180 in Wu. The English participants generated 270 English tokens, the Mandarin speakers 630 tokens (270 English +360 Mandarin), and the Mandarin/Wu speakers 810 tokens (270 English+360 Mandarin +180 Wu).

#### Procedure and measurements

Each participant was recorded individually in the soundproof lab with an experimenter present, and they were informed that their recordings were private, confidential, and unrelated to academic credit. The recordings were made using a Shure PG42 Side Address Condenser Microphone with a frequency response between 20 and 20,000 Hz, and a non-linear, steeply decreasing frequency response above 15,000 Hz. The microphone was connected to an M-Audio Audiophile USB soundcard attached to a laptop computer running Cool Edit Pro 2.0 software. All recordings were made at a sampling rate of 22,050 Hz, 16 bit. Before the experiment, the participants were asked to confirm their knowledge of any words they had trouble pronouncing. Then they were asked to pronounce each sentence clearly at a moderate speech rate. The target sentences were presented in PowerPoint slides, with one sentence per slide in random order and no stimulus appeared more than twice in a row. No inter-stimulus interval was set. For each individual English, Mandarin, and Mandarin/Wu speaker, the recording lasted approximately 10, 15, and 20 minutes, respectively. The recording sessions were spread out across several days to avoid experimenter fatigue.

Measurements of fricatives were made using Praat (Boersma and Weenink, 2018). Fricative onset was defined as the point at which high-frequency energy first appeared on the spectrogram, whereas fricative offset was identified as the intensity minimum immediately preceding the onset of the vowel periodicity. For the spectral moment analysis, an FFT spectrum was made over a 40-ms full Hamming window placed in the middle of the frication noise.

#### Acoustic features

Studies on fricatives have concentrated on several attributes: amplitude, duration, spectral properties and fricative-vowel transitional characters. Among these parameters, the spectral properties play an important role in identifying the place and manner of fricative articulation (McMurray and Jongman, 2011; Spinu and Lilley, 2016; Redmon and Jongman, 2018; Chodroff and Wilson, 2020; Mellesmoen and Babel, 2020; Beristain, 2021; Rao and Shaw, 2021). Spectral properties include spectral characteristics of fricative noise and the fricative-vowel transitional portion. Specifically, the former is described by the term ‘spectral moments’, i.e., the “mean,” “variance,” “skewness” and “kurtosis” of the spectral energy distribution within the duration of the fricative (Jongman et al., 2000; Chiu et al., 2020). The spectral moments capture a great deal of information on the frication noise: Spectral mean and variance reflect the average energy concentration and energy range (dispersion ratio), respectively, at a given time. Particularly, the apical fricatives have a larger energy range than the laminal ones, because the former involves looser contact of the tongue tip and palate (Butcher, 2015). By contrast, spectral skewness and kurtosis are indicators of the energy distribution’s asymmetry and peakedness, respectively, within that time. Researchers find that spectral mean and skewness are negatively correlated with the length of the front resonating cavity, whereas spectral variance or kurtosis indicates whether the tongue posture is apical or laminal (Li et al., 2009).

In the current study, the vowels that follow the fricatives are not entirely the same for different languages (*cf.* section 1.2), therefore we disregarded the fricative-vowel transitional properties and only considered the spectral traits for the noise part.

### Results

#### Spectral value

Spectral value for English and Chinese post-alveolar fricatives, as well as those realized in the ‘accented’ speech, is presented in Tables 4, 5. Mandarin/Wu /ɕ/ had the highest mean value, suggesting the small length of the front resonating cavity was formed by the advanced tongue body. Mandarin /ʂ/ was close to English /ʃ/ in spectral mean (4,606 Hz vs. 4,268 Hz), indicating that locations of the tongue body were similar for these two segments. A rather large variance value (2,482 Hz) for Mandarin /ʂ/ further indicated the loose contact between the tongue tip and the palate. The palatal /ɕ/ had a shorter front resonating cavity than the retroflex or post-alveolar fricatives, which negatively correlated with its greater spectral mean value (7,354 Hz in the current study) than those of the other fricatives. Mandarin /ɻ/ was produced with a particularly low spectral mean and variance but markedly high skewness and kurtosis value, perhaps because they were produced with greater vocal cord vibration than average fricatives, which drove down its spectral mean value, skewing the spectra and increasing its kurtosis. On top of that, the voicing of /ɻ/ may generate enhanced resistance to airflow from the lungs, and thus pull down the degree of air turbulence (spectral variance).

**Table 4:** Mean values of the spectral moments for English, Mandarin, Mandarin-Wu and the accented voiceless post-alveolar fricatives.

Articulation	Mean (Hz)	Variance (Hz)	Skewness	Kurtosis
English /ʃ/	4,268	1,379	1.079	2.317
Mandarin /ʂ/	4,606	2,482	0.629	0.751
Mandarin/Wu /ɕ/	7,354	1975	−0.679	0.201
Mandarin-accented /ʃ/	4,701	2,103	0.773	0.875
M/Wu-accented /ʃ/	5,842	1874	−0.026	0.315

**Table 5:** Mean spectral parameter values for English, Mandarin, Mandarin-Wu and the accented voiced post-alveolar fricatives.

Articulation	Mean (Hz)	Variance (Hz)	Skewness	Kurtosis
English /ʒ/	3,068	1806	0.838	3.011
Mandarin /ɻ/	390	472	11.359	339.39
Wu /ʑ/	2,229	1,243	5.926	99.684
Mandarin-accented /ʒ/	415	430	11.611	262.24
M/Wu-accented /ʒ/	5,862	1990	−0.064	0.699

Statistical analysis showed that Mandarin and Mandarin/Wu speakers did not differ in the production of /ʂ/, /ɕ/ and /ɻ/ in terms of four spectral parameters, so we collapsed these segments that were produced by Mandarin and Mandarin/Wu speakers into a single category.

As there were three speaker groups in the study and the assumptions of normality and homogeneity of variance were violated in the parametric statistics, we performed the Kruskal-Wallis test to look into the differences between each group. It turned out that there was a significant difference between native and accented English fricatives (Table 6). The *post-hoc* analysis showed Mandarin- and Mandarin/Wu-accented /ʃ, ʒ/ were statistically different from the target /ʃ, ʒ/ in all spectral moments, and Mandarin and Mandarin/Wu-accented outputs differed from each other too.

**Table 6:** Results of the Kruskal-Wallis test on the spectral moments for English native, Mandarin- and Mandarin/Wu-accented post-alveolar fricatives.

Main effect for /ʃ/	Mean	Variance	Skewness	Kurtosis
	H = 129.211 (*η^2^ = 0.32*) df = 2 *p* < 0.001	H = 203.882 (*η^2^ = 0.5*) df = 2 *p* < 0.001	H = 99.328 (*η^2^ = 0.24*) df = 2 *p* < 0.001	H = 86.512 (*η^2^ = 0.21*) df = 2 *p* < 0.001
Turkey *post hoc*	Mean	Variance	Skewness	Kurtosis
English /ʃ/ vs. Mandarin-accented /ʃ/	*p* < 0.01	*p* < 0.001	*p* < 0.05	*p* < 0.001
English /ʃ/ vs. M/Wu-accented /ʃ/	*p* < 0.001	*p* < 0.001	*p* < 0.001	*p* < 0.001
Mandarin-accented /ʃ/ v.s. M/Wu-accented /ʃ/	*p* < 0.001	*p* < 0.001	*p* < 0.001	*p* > 0.05
**Main effect for /ʒ/**	**Mean**	**Variance**	**Skewness**	**Kurtosis**
	H = 321.333 (*η^2^ = 0.79*) df = 2 *p* < 0.001	H = 274.987 (*η^2^ = 0.68*) df = 2 *p* < 0.001	H = 266.009 (*η^2^ = 0.66*) df = 2 *p* < 0.001	H = 280.606 (*η^2^ = 0.69*) df = 2 *p* < 0.001
Turkey *post hoc*	Mean	Variance	Skewness	Kurtosis
English /ʒ/ vs. Mandarin-accented /ʒ/	*p* < 0.001	*p* < 0.001	*p* < 0.001	*p* < 0.001
English /ʒ/ vs. M/Wu-accented /ʒ/	*p* < 0.001	*p* < 0.05	*p* > 0.05	*p* < 0.001
Mandarin-accented /ʒ/ vs.M/Wu-accented /ʒ /	*p* < 0.001	*p* < 0.001	*p* < 0.001	*p* < 0.001

While both Mandarin-accented /ʃ/ and Wu-accented /ʃ/ were different from native English /ʃ/, the spectral parameter values showed that Mandarin-accented /ʃ/ was generally closer (than Wu-accented /ʃ/) to native English /ʃ/. Mandarin-accented /ʃ/ was more likely to be produced with the tongue further back (as seen in the comparatively lower spectral mean) and being apical, since the greater variance ratio indicated less constriction between the tongue and the palate. By contrast, Mandarin/Wu-accented /ʃ/ was produced with the tongue rather fronted (with a much higher spectral mean than the target /ʃ/). As for /ʒ/, the Mandarin-accented production and the Mandarin/Wu-accented production differed from native English /ʒ/ in different directions: Mandarin-accented /ʒ/ exhibited a significantly lower spectral mean and variance but a rather higher skewness and kurtosis when compared to the target /ʒ/; for Mandarin/Wu-accented /ʒ/, the pattern in the spectral moments is exactly the opposite of that of Mandarin-accented /ʒ/.

#### Features of the accented fricatives in relation to L1

A Linear Discriminant Analysis (LDA) was conducted to examine the relations between the accented L2 outputs and the L1 equivalents. All four spectral parameters were included as independent variables in two LDA models: in Model 1, the dependent variables included the accented /ʃ/ and its L1 counterparts, namely, Mandarin /ʂ/, /ɻ/, and Mandarin/Wu /ɕ/; in Model 2, the accented /ʒ/ and the same L1 counterparts were included.

Overall, 64.5% of the data in Model 1 and 58.6% of the data in Model 2 were correctly classified; for the two models, the strongest correlates were ‘mean’-‘variance’-‘skewness’ and ‘mean’-‘variance’-‘kurtosis’, respectively. Categorization of the accented fricatives predicted by the LDA is shown in Tables 7, 8. The percentages represent the rate at which the accented fricatives were categorized into a certain group.

**Table 7:** Categorization of the accented /ʃ/.

	English /ʃ/	Mandarin /ʂ/	Mandarin /ɻ/	Wu /ɕ/	Mandarin -accented /ʃ/	M/Wu-accented /ʃ/
Mandarin-accented /ʃ/	14.1	**33.3**	0	2.2	33.3	17
M/Wu-accented /ʃ/	13.2	11	0	**26.5**	14.7	34.6

The bold percentages indicate the highest correct classification rate category (other than the categories in question) in participants’ L1 phonetic system. Predicted group membership (%).

**Table 8:** Categorization of the accented /ʒ/.

	English /ʃ/	Mandarin /ʂ/	Mandarin /ɻ/	Wu /ɕ/	Mandarin –accented /ʒ/	M/Wu-accented /ʒ/
Mandarin-accented /ʒ/	1.5	0	**59.6**	0	39	0
M/Wu-accented /ʒ/	11.9	23	0	**30**	0	35.6

The bold percentages indicate the highest correct classification rate category (other than the categories in question) in participants’ L1 phonetic system. Predicted group membership (%).

The LDA analysis showed that a number of Mandarin-accented /ʃ/ and /ʒ/ overlapped with Mandarin /ʂ/ and /ɻ/, respectively, whereas a considerable portion of Mandarin/Wu-accented /ʃ/ and /ʒ/ overlapped with Mandarin/Wu /ɕ/.

**Figure 1:**
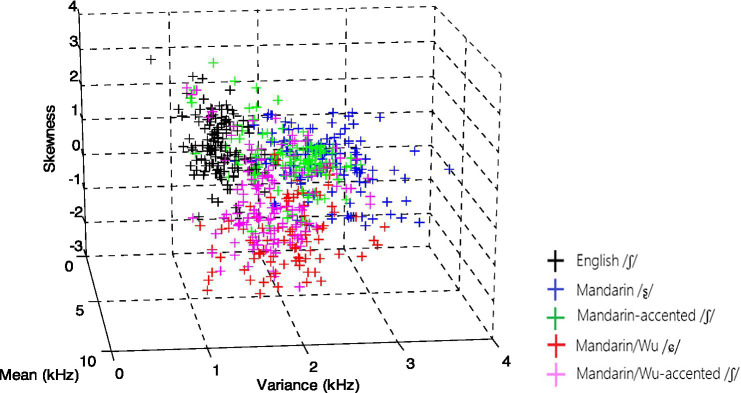
Acoustic space of English /ʃ/, Mandarin /ʂ/, Mandarin/Wu /ɕ/, Mandarin- and Mandarin/Wu-accented /ʃ/.

**Figure 2:**
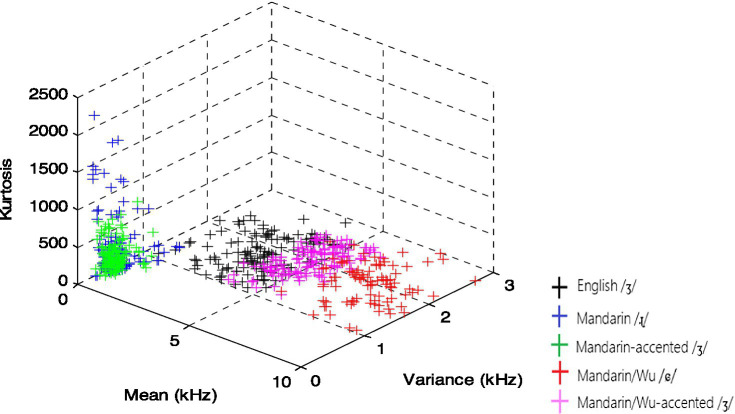
Acoustic space of English /ʒ/, Mandarin /ɻ/, Mandarin/Wu /ɕ/, Mandarin- and Mandarin/Wu-accented /ʒ/.

The following figures displayed essential variables in the LDA analysis. The overlap between Mandarin-accented /ʃ/ and Mandarin /ʂ/ was evident in Figure 1, as were the numerous tokens between Mandarin/Wu-accented /ʃ/ and Mandarin/Wu /ɕ/. On the other hand, in Figure 2, the Mandarin-accented /ʒ/ appeared to totally overlap with Mandarin /ɻ/, whereas Mandarin/Wu-accented /ʒ/ partially overlapped with Mandarin/Wu /ɕ/.

**Figure 3:**
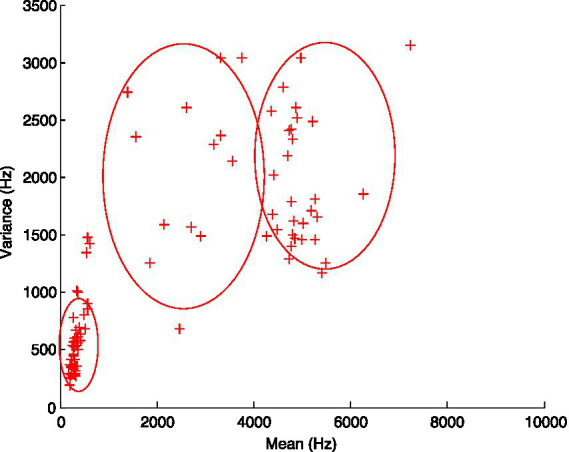
Articulatory space of /ʑ/ in Wu.

In the current study, the voiced /ʑ/ in Wu was excluded from all statistical analysis, because there were large individual differences in the production of /ʑ/ among Mandarin/Wu speakers, and no regular pattern was found in the value of all four spectral moments. The spectral values of /ʑ/ in the articulatory space were widely dispersed in the acoustic space (‘spectral variance’ 200–3,000 Hz as a function of ‘spectral mean’ 300–7,500 Hz, see Figure 3). The inconsistent value of /ʑ/ supported the claim that the voiced fricative /ʑ/ in Wu has been subject to synchronic change.

### Discussion

Neither Mandarin- nor Mandarin/Wu-accented /ʃ, ʒ/ were native-like, so transfer from these speakers’ L1 equivalents was very likely to occur when English /ʃ, ʒ/ were targeted. Table 9 listed the ‘substitution’ patterns that Mandarin and Mandarin/Wu speakers employed when pronouncing the targets. Admittedly, the ‘substitutions’ were not pure L1 fricatives, since most accented segments were spectrally somewhere between L2 targets and L1 counterparts. The more accurate description of the caption ‘substitution’ in this table could be “/ʂ/−like,” “/ɻ/−like” or “/ɕ/−like” phonemes. However, as the LDA statistically categorized the accented L2s into the L1 equivalents to a significant degree, we continued to refer to the substitutions as /ʂ/, /ɻ/ and /ɕ/, respectively.

**Table 9:** Substitution of the English post-alveolar fricatives by Mandarin and Mandarin/Wu speakers.

Speakers	Target articulation	Substitution
Mandarin	English /ʃ/	/ʂ/
	English /ʒ/	/ɻ/
Mandarin/Wu	English /ʃ/	/ɕ/
	English /ʒ/	/ɕ/

Unlike Mandarin monolinguals, who substituted English /ʃ/ and /ʒ/ with Mandarin /ʂ/ and /ɻ/, the Mandarin-Wu bilinguals employed /ɕ/ for both /ʃ, ʒ/, indicating these bilinguals treated the two languages (Mandarin vs. Wu) differently. Although Mandarin was acquired at an early age and was proficiently used by Mandarin/Wu speakers, their production of L2 segments was not the same as Mandarin speakers.

It has been suggested that exemplars of different languages are kept in a single, unified mental map (Hall, 2008; Wei, 2019). Here we proposed that the Mandarin and Wu language systems were entrenched at different depths of the unified mental map, and therefore functioned as two distinct systems that did not interact in the learning process of a new language. In the current experiment, the phonetic system of L1 Wu may play a dominant role in the acquisition of English phonetics by Mandarin/Wu speakers.

In the next experiment, we examined native English listeners’ assessment on the accented productions for a better understanding of how well the target segments were uttered.

## Rating experiment

Accent ratings were first predicted based on the acoustic features obtained in the preceding experiment. An LDA study taking native English- and accented−/ʃ, ʒ/ as dependent variables suggested that Mandarin-accented /ʒ/ might be rated as most accented because it was least likely to be categorized as native English /ʒ/ (1.5%). Mandarin/Wu-accented /ʒ/ (8.1% as English /ʒ/), Mandarin/Wu-accented /ʃ/ (10.4% as English /ʃ/) and Mandarin-accented /ʃ/ (11.1% as English /ʃ/) were then categorized as the target sounds in ascending order, indicating that they might be rated as comparatively less accented. However, it wasn’t clear if there was any real difference between these percentages as they were very close to one another.

Next, a rating experiment was conducted to see how native English speakers perceived the degrees of accentedness of Mandarin- or Mandarin/Wu accented /ʃ, ʒ/. In the rating test, the raters listened to stimuli read by native English, Chinese Mandarin or Mandarin/Wu speakers, and decided the degrees of accentedness of each stimulus.

### Methods

#### Stimuli

A total number of 72 Mandarin-accented, Mandarin/Wu-accented and native /ʃ/ and /ʒ/ productions from 15 Mandarin, 15 Mandarin/Wu and 12 English native speakers in experiment 1 served as the stimuli. Two stimuli of accented sounds were randomly selected from each Chinese speaker, both voiced and voiceless. Since the stimuli pronounced by English native speakers served as fillers, only one stimulus was chosen from each of the 12 English natives. All stimuli were adjusted to the same amplitude.

#### Raters

The raters were four native speakers of English who were also phoneticians or had experience in teaching English as an L2 to Chinese learners: two of them were PhDs in linguistics from the University of Indiana, and the other two were ESL teachers at Tongji University (Shanghai). All reported normal hearing and had a good knowledge of articulatory phonetics.

#### Procedure

Consonant rating can be challenging in the absence of a segmental context, but it can also be easily influenced by contexts (other segments) within the same word. Therefore, we decided to split the rating test into two sessions, each held on different days. In the first session, the raters were asked to pay proper attention to the phonemes /ʃ/ or /ʒ/ in each word, and assign specific ratings for this phoneme’s quality on a scale from 1 (no foreign accent) to 7 (very strong foreign accent). In the second session, the raters were asked to attend to the general accentedness of each word and assign an overall pronunciation rating (also from 1 to 7) to it. Results of the two sessions were compared to see if there was any difference in the patterns of segment and word ratings, which can be an indication as to whether raters have been focused on specific segments when instructed to do so.

In each session, the raters were asked to listen to the speech material in a quiet room, and assign scores individually. Target stimuli and fillers were randomized and delivered on a computer using E-prime 2.0 (Schneider et al., 2002) with high-quality headphones (Sennheiser HDA200). During the experiment, the raters could adjust the volume to a comfortable level.

In a familiarization phase, 10 words spoken by speakers with different L1 backgrounds were selected to both familiarize the raters with the task and stabilize their ratings. In the formal rating test, 72 English words were rated with an inter-stimulus interval of 6,000 ms. Each session lasted approximately 18 min.

### Results

**Figure 4:**
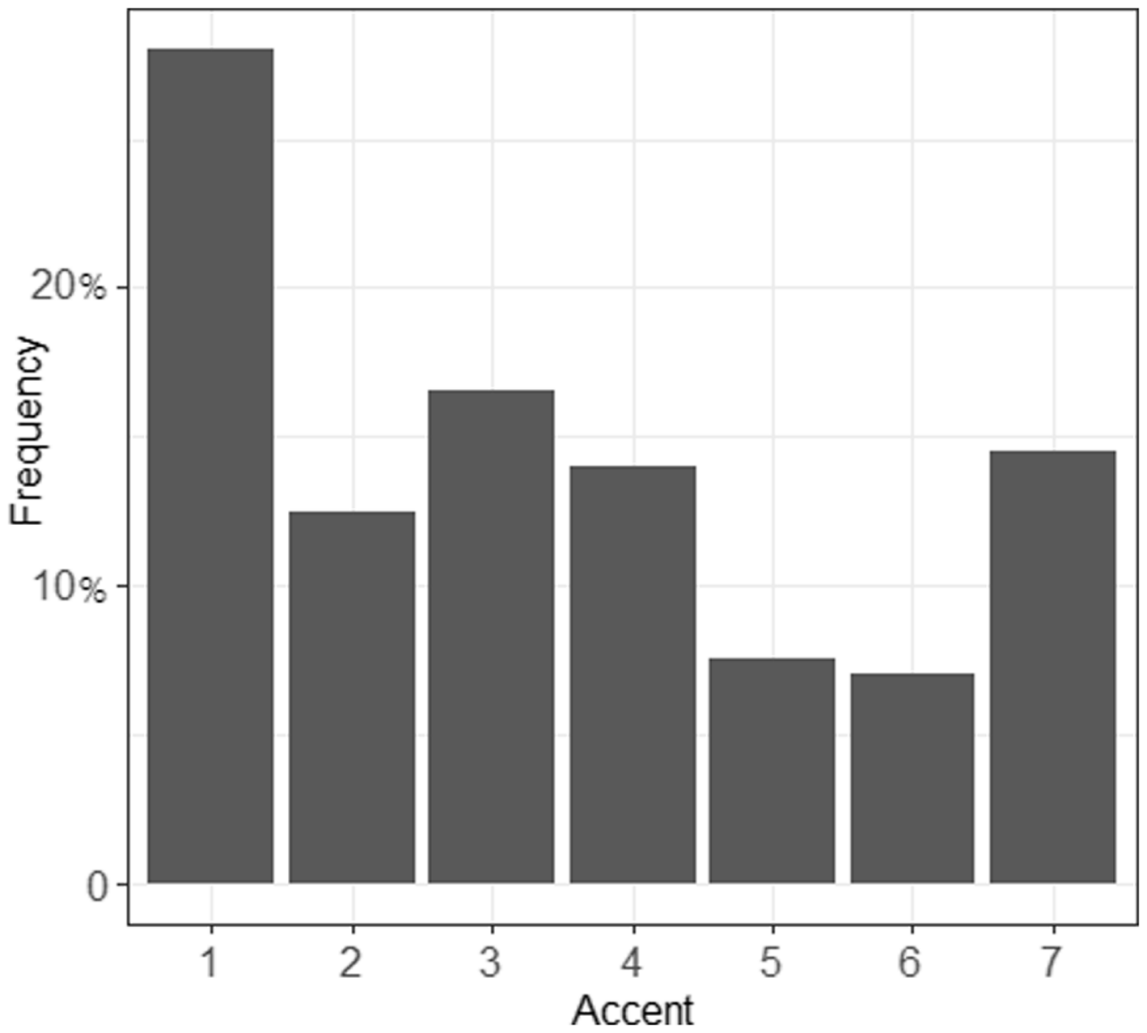
Accent of the segments /ʃ/ and /ʒ/ (section 1). 1 = no foreign accent, 7 = very strong foreign accent.

**Figure 5:**
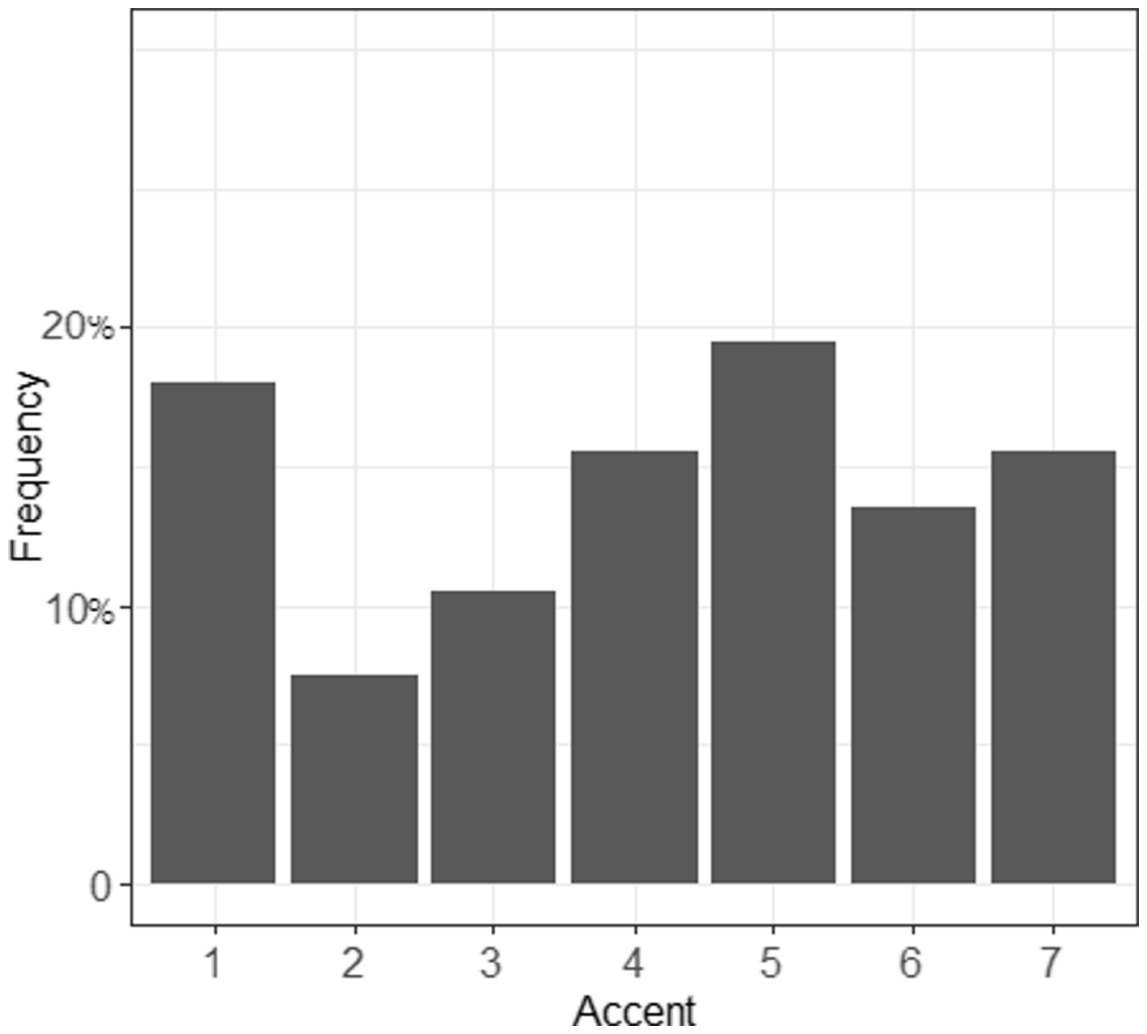
Accent of the words (containing /ʃ/ and /ʒ/, section 2). 1 = no foreign accent, 7 = very strong foreign accent.

A Krippendorff’s α of 0.846 indicated a high level of consistency across the four raters for both sessions. However, inter-session comparison revealed a different pattern between phoneme and word ratings (*cf.* Figures 4, 5): for session 1, more than 25% of the judgments were given a score of 1, which denoted no foreign accent, while for session 2, this number was 18%. In general, segments were less likely to be rated with an accent than words that contained these segments.

**Figure 6:**
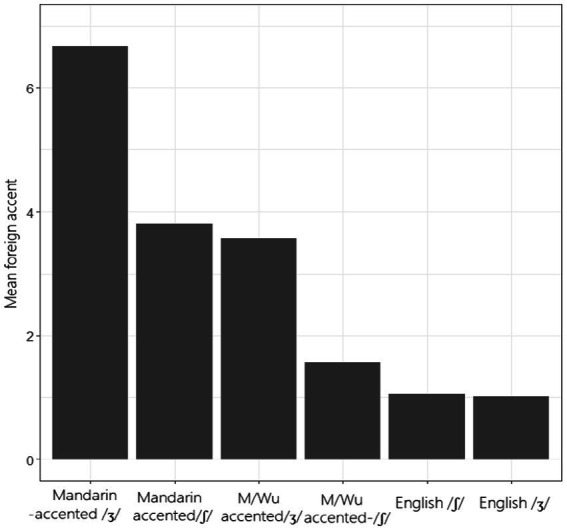
Mean ratings on individual segment given by native English listeners.

Ranking of the accent for individual segments is shown in Figure 6. The mean perceived ratings of the accentedness ranged from 1.0 (native /ʃ/) to 6.67 (Mandarin-accented /ʒ/). Mandarin-accented /ʒ/ had the highest accented score, suggesting that native English speakers found this group to be the most accented; after that were Mandarin-accented /ʃ/ and Mandarin/Wu-accented /ʒ/, with scores of 3.57 and 3.8, respectively; the rating for Mandarin/Wu-accented /ʃ/ was rather low (1.57) and was comparable to native English speakers (1.06). To further examine the differences, we ran a Linear Mixed Effect model (with repeated effects in raters), and found a significant main effect in ‘type of segment’ [*F* (5, 58.25) = 134.034, *p* < 0.001]. Subsequent pairwise comparisons revealed that the scores between most groups of the accented sounds were significantly different (*p* < 0.001), with the exception of the scores between Mandarin-accented /ʃ/ and Mandarin/Wu-accented /ʒ/, and the scores between Mandarin/Wu-accented /ʃ/ and native /ʃ, ʒ/. The rankings of these accent scores only partially matched the prior-test predictions, with Mandarin-accented /ʒ/ being the most accented one.

The Pearson correlation coefficient between accent ratings and English proficiency levels (CET-4 scores) was −0.067 (*p* = 0.7) for individual speakers, indicating that the general English levels had little effect on the accent of the two groups of participants.

### Interpretation of the rated accentedness

Results of the current experiment revealed that the target /ʒ/ uttered by Mandarin speakers sounded the most accented to native English listeners, while the target /ʃ/ generated by Mandarin/Wu speakers was assessed to be of no accent. Chinese learners of English may substitute the target L2 with the perceived closest L1 segments, but native English listeners interpreted the closeness of these substitutions differently from L2 learners. A Random Forest analysis exploring the influence of the spectral cues on the rating outcomes revealed that ‘spectral mean’ and ‘spectral skewness’ were the two most important variables in the regression (Gini importance for the two variables was 55.6% in total). This suggests that English listeners might be especially sensitive to tongue position (i.e., length of the front resonating cavity, Li et al., 2009) when assessing the segments produced. To identify what factors may be responsible for the degrees of rated accentedness in each fricative type, we conducted a general comparison of the data in the production and rating tests and discovered that phonological factor may play a role in how the L2 output was assessed.

**Figure 7:**
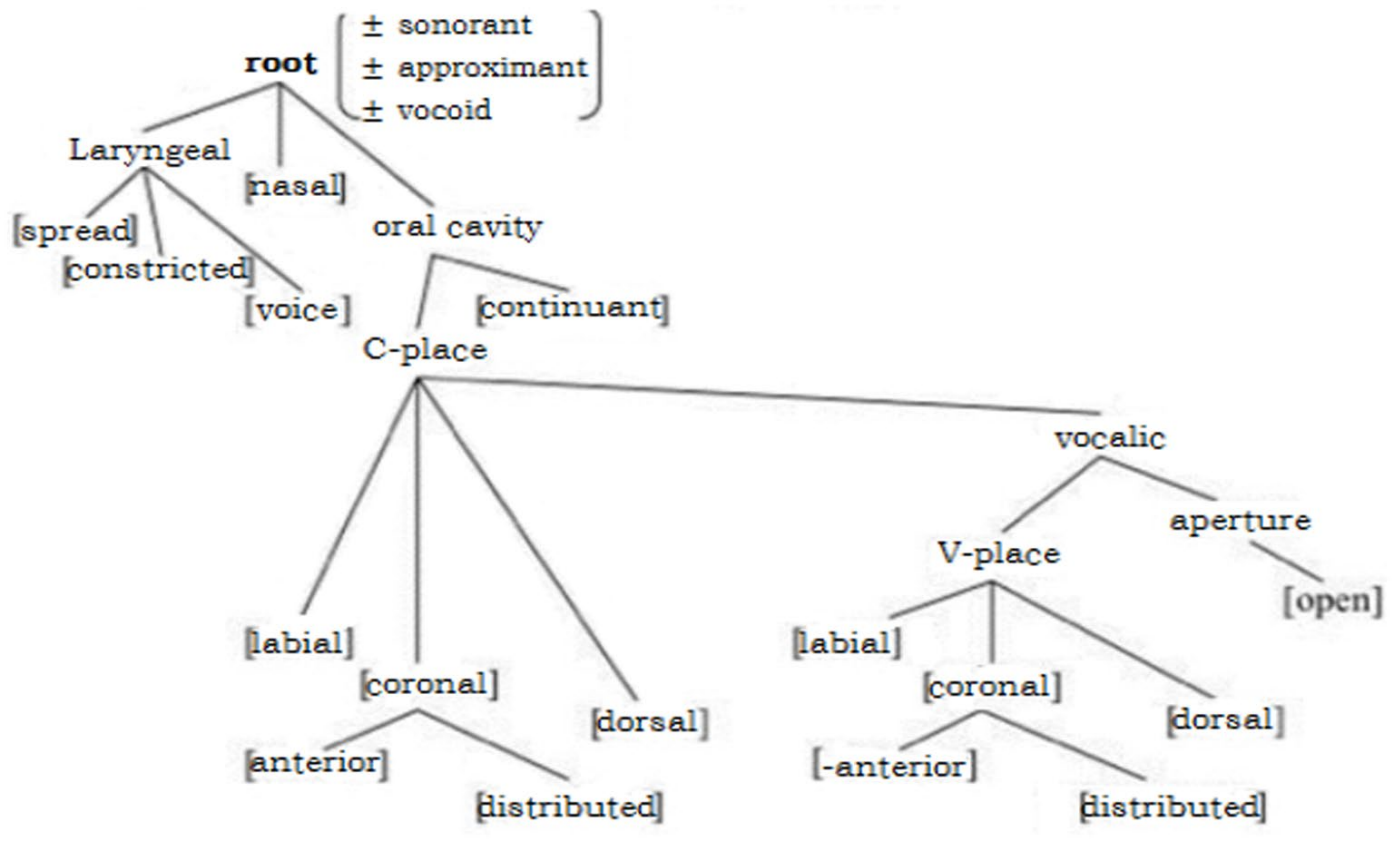
Phonological feature geometry (Clements and Hume, 1995).

Phonological features grouped into different categories (major class features, laryngeal features, manner features, and place features) may affect accent rating, in that sensitivity to the accent could derive from ‘feature boundary violation’. Researchers have proposed a ‘phonological feature geometry’ (Figure 7), in which a higher order in the feature hierarchy would correspond to a heavier feature weight (LaCharité and Prévost, 1999; Mah and Archibald, 2003; De Jong and Hao, 2018; Jones and Schnupp, 2021), and an ‘L1-L2 feature boundary violation’ regarding articulator node would be more difficult to acquire than a terminal node. This indicates that the greater weight on the ‘L1-L2 feature boundary violation’, the more likely that a non-native substitution is dissimilar from the target segment from the native listeners’ perspective, and will therefore be judged as heavier accented.

In the current research, a substitution that violated a feature in the root node [±approximant] of the phonological hierarchy (Mandarin /ɻ/ vs. English /ʒ/) may generate a strong accent in the perception of English native listeners, whereas a substitution that violated features in a terminal node (“±voiced” for Wu /ɕ/ to English /ʒ/ and ‘±distributed’ for Mandarin /ʂ/ to English /ʃ/) may be rated as having a medium level of accent. Here we assigned weight ‘0’ to the feature violation of substituting /ʃ/ with /ɕ/, as both /ɕ/ and /ʃ/ were defined as ‘–anterior, +distributed’ in their respective phonological systems; by contrast, the retroflex /ʂ/ was different from /ʃ/ in that /ʂ/ was ‘–distributed’ and /ʃ/ ‘+distributed’, the degree of the feature violation in this situation was ‘1’. Similarly, in the case of replacing /ʒ/ by /ɕ/, the low-ranked place and laryngeal feature were violated, so a weight ‘1’ in feature violation was expected; in the condition of substituting the fricative /ʒ/ with an approximant /ɻ/, both the high-rank feature ‘manner’ (which makes a weight ‘2’ feature violation) and place boundary (±distributed) got crossed, which amounted to a degree ‘3’ feature violation (see Table 10).

**Table 10:** Weights of feature violation correspond to different substitution strategies.

Substitution	Feature violation	Weight
/ɕ/ to /ʃ/	PoA (0)	0
/ʂ/ to/ʃ/	PoA (1)	1
/ɕ/ to /ʒ/	Lar (1)	1
/ɻ/ to /ʒ/	PoA(1) + MoA(2)	3

PoA and MoA stand for Place of Articulation and Manner of Articulation, respectively.

Based on the gradient accent rating by English native speakers, we tentatively propose that phonological features in the English speech sound system may vary in weight on two grounds: first, the manner features weigh more than place features and laryngeal features (voice) because of their higher position in the phonological hierarchy; second, within the dimension of place features, there may be varying degrees of weight based on the hierarchies of the feature nodes. In the current study, the more weights assigned to ‘feature boundary violation’, the more probable it was that English native listeners would judge the substitution made by Chinese learners of English as having a heavy accent.

## General discussion

In the current study, we investigated the production of the English post-alveolar fricatives /ʃ, ʒ/ by Chinese Mandarin- and Mandarin/Wu speakers. We examined the spectral moments of the accented /ʃ, ʒ/ and compared them with that of the target sounds by referring to the L1 equivalents. Mandarin and Mandarin/Wu speakers produced the target /ʃ, ʒ/ in distinct ways: Mandarin speakers’ production of /ʃ/ and /ʒ/ exhibited features of Mandarin retroflex /ʂ/ and /ɻ/, respectively, whereas Mandarin/Wu speakers’ output for both /ʃ/ and /ʒ/ showed features of /ɕ/. The findings were generally consistent with our research expectations, with the exception of Wu /ʑ/, which did not seem to play a significant role in L2 production. Results of the subsequent rating test revealed that native English listeners scored Mandarin/Wu-accented /ʃ/ as having no accent and Mandarin-accented /ʒ/ as a heavy accent, with the other accented sounds (Mandarin-accented /ʃ/, Mandarin/Wu-accented /ʒ/) being ranked somewhere in the middle. Phonological considerations may help to explain the degree of perceived accentedness in L2 production. As mentioned earlier, many theories pertaining to non-native speech perception and production have proposed that the L1 segments have a significant impact on L2 segment learning. This appears to be the case in the current study: Mandarin monolinguals projected the English post-alveolar /ʃ, ʒ/ to Mandarin retroflex /ʂ, ɻ/, whereas Mandarin/Wu speakers, though early bilinguals, performed differently, perhaps because the Mandarin-specific phonemes do not play a dominant role in their phonological systems.

The speech learning model (SLM) highlights the importance of L1-L2 dissimilarity to L2 sound learning, in that L1-L2 distance, which is most likely perceived from the learners’ perspective, is an important factor in predicting the learning results of the L2 segment (i.e., formation the L2 category). Findings of the current research indicated that this ‘L1-L2 dissimilarity’ should be considered, however, from two different perspectives: that of Chinese learners of English and that of native English listeners, both of which can be important in determining how well the target L2 sound is learnt. Chinese learners of English may perceive the ‘L1 vs. L2’ dissimilarity in a way entirely different from native English listeners’ perception of the likeness of the ‘non-native substitutes vs. English segments’. For example, due to what they may perceive as similar between the two languages, Mandarin speakers replaced English /ʒ/ for Mandarin /ɻ/, but this /ʒ/−/ɻ/ proximity was not well accepted by native English listeners. In fact, they found it difficult to take the approximant /ɻ/ as a substitute for fricative /ʒ/ and thus rated it as having ‘high degree of accent’. Compared with that, the same group of English listeners were not sensitive to the non-native /ɕ/, which was used to substitute the target /ʃ/ by Mandarin/Wu speakers. Native listeners and L2 learners of a specific language may perceive a ‘nonnative-native pair’ differently (e.g., L2 learners’ L1 vs. L2 could be native listeners’ L2 vs. L1, depending on each party’s point of view) for two reasons: (1) Each group has a separate phonological structure in its L1, so the specific sound may be classified differently and consequently have different weight within each structure. (2) Different types of learners/listeners are involved when an L2 sound is produced and evaluated. The Chinese learners of English were the typical ‘L2 learners’ in the current study, their perception of the ‘nonnative-native pair’ was likely to differ from the native English raters who were the ‘naïve listeners’ (without prior knowledge of Chinese), because the lexical learning may exert an additional influence on the L2 learners’ perception of the nonnative sounds, but never on ‘naïve listeners’.

Whatever factors may contribute to the divergent perception patterns between L2 learners and native listeners, both parties’ perception can be crucial when determining the learning outcomes. For example, if an L2 segment is close to its L1 counterpart, the L2 learners may have trouble distinguishing the subtle difference between the two and thus struggle to build the L2 category with more effort. However, how well it is actually acquired may involve other factors aside from this, depending on whether native listeners of this language also perceive this ‘non-native substitute--native segment’ pair to be equally similar (or dissimilar): if so, then the native listeners could not discern the difference either and thus determine the L2 segment to be well acquired (e.g., the rating of /ɕ/ as the substitution of target /ʃ/ in the current study). In other words, small L1-L2 dissimilarity does not necessarily result in poor acquisition if this difference is also small from native listeners’ perspective. For the same reason, great L1-L2 dissimilarity does not always yield any ideal result when native listeners of the L2 segment are pretty sensitive to all the subtle aspects of the difference (e.g., the rating of /ɻ/ as substitution of English /ʒ/ in the current study).

The SLM theory emphasizes the importance of L2 learners’ perceived L1-L2 dissimilarity in the prediction of the L2 learning result, however, the ‘non-native substitute - native segment’ dissimilarity as perceived by native English listeners also has an impact on defining how well the target fricatives were learned in the current study. Hence, taking into account both perspectives of L2 learners and native listeners of a specific language is necessary when evaluating the learning outcome of an L2 segment; otherwise, we may run the risk of failing to see the complete picture of the matter.

## Limitations

One limitation of the current study is its limited sample size in the production and rating experiments. Though the sample in the production experiment was comparable to some previous studies in which acoustic properties of fricatives between different groups were investigated and compared (Jones and Llamas, 2008; Li et al., 2009), the study could benefit more from a larger sample size. Another issue is that the listeners in the rating test of the current study were all native English speakers with linguistic knowledge. Whether linguistically knowledgeable raters are equivalent to untrained native speakers needs further investigation. In the future, accent evaluation will be conducted by inviting ordinary native English speakers to a larger population follow-up study.

## Data availability statement

The data supporting the conclusions of this article are available from the corresponding author upon reasonable request.

## Ethics statement

The studies involving human participants were reviewed and approved by the Ethical Committee of Ningbo University of Technology. Written informed consent for participation was not required for this study in accordance with the national legislation and the institutional requirements.

## Author contributions

WC designed the study, analyzed the data, and wrote the manuscript. JW helped in the analysis of the data and was also involved in writing the manuscript. Both authors contributed to the article and approved the submitted version.

## Conflict of interest

The authors declare that the research was conducted in the absence of any commercial or financial relationships that could be construed as a potential conflict of interest.

## Publisher’s note

All claims expressed in this article are solely those of the authors and do not necessarily represent those of their affiliated organizations, or those of the publisher, the editors and the reviewers. Any product that may be evaluated in this article, or claim that may be made by its manufacturer, is not guaranteed or endorsed by the publisher.
